# Flavonoids in the treatment of *Leishmania amazonensis*: a review of efficacy and mechanisms

**DOI:** 10.3389/fphar.2025.1642005

**Published:** 2025-08-07

**Authors:** Vinicius Lopes Lessa, Guilherme Drescher, Gustavo Gonçalves, João Carlos Baptista Lopes, Rafael Felipe da Costa Vieira, Fabiano Borges Figueiredo

**Affiliations:** ^1^ Carlos Chagas Institute, Oswaldo Cruz Foundation (Fiocruz), Curitiba, Paraná, Brazil; ^2^ Graduate Program in Veterinary Sciences, Federal University of Paraná, Curitiba, Paraná, Brazil; ^3^ Graduate Nursing Progam, Campos de Andrade University Center, Curitiba, Paraná, Brazil; ^4^ Department of Epidemiology and Community Health, College of Health and Human Services, University of North Carolina at Charlotte, Charlotte, NC, United States; ^5^ Center for Computational Intelligence to Predict Health and Environmental Risks (CIPHER), University of North Carolina at Charlotte, Charlotte, NC, United States

**Keywords:** natural compounds, flavonoids, *Leishmania amazonensis*, *in vitro* assays, treatment

## Abstract

Leishmaniasis is caused by protozoan parasites of the genus *Leishmania*. In recent years, natural compounds have attracted significant interest due to their potential efficacy and lower toxicity compared to synthetic chemical compounds. This review analyzed studies retrieved from the PubMed and Google Scholar databases, focusing on the use of flavonoids against *Leishmania amazonensis*. Only studies testing flavonoids with known activity against the parasite were included and categorized according to their leishmanicidal efficacy. Based on the criteria established to identify the most comprehensive studies, 52 were included in the analysis. Of these, three studies met at least 13 of the evaluation parameters (70%) and were considered the most complete. Analysis of IC_50_ values reported in these articles revealed the activity of 69 flavonoids. Among the assays on amastigote forms, 33 reported high activity, and six reported moderate activity. For assays on promastigote forms, 32 experiments reported high activity, 16 showed moderate activity, and two demonstrated weak activity. Of the flavonoids tested, morelloflavone-4‴O-β-D-glycosyl and pinostrobin showed the highest activity, while naringenin exhibited the weakest activity, specifically against promastigote forms. In the cytotoxicity assays, carajurin and luteolin exhibited the highest selectivity indices reported in the articles. This review emphasizes the importance of studying flavonoids, particularly those extracted from plants and propolis, to advance our understanding and treatment of *L. amazonensis* infections.

## 1 Introduction

Leishmaniasis, caused by protozoan parasites of the genus *Leishmania*, is a significant public health concern affecting millions of people worldwide. Over one billion individuals are at risk of contracting leishmaniasis due to living in endemic regions (Steverding, 2017). Every year, an estimated 30,000 new cases of visceral leishmaniasis (VL) and over one million new cases of cutaneous leishmaniasis (CL) are reported (Steverding, 2017; Burza et al., 2018; WHO, 2023).

*Leishmania* species are typically divided into two primary groups: Old and New World species. The Old-World species are found in Africa, Asia, the Mediterranean region, and the Middle East and include *Leishmania tropica*, *Leishmania major*, *Leishmania aethiopica*, and *Leishmania donovani* (Hassan et al., 2022; Alemayehu and Alemayehu, 2017). The New World species, which are endemic to the Americas, include *Leishmania mexicana*, *Leishmania amazonensis*, *Leishmania braziliensis*, *Leishmania panamensis*, *Leishmania peruviana*, *Leishmania guyanensis*, *Leishmania pifanoi*, *Leishmania venezuelensis*, *Leishmania shawi*, and *Leishmania lainsoni* (Hassan et al., 2022; Alemayehu and Alemayehu, 2017).

Leishmaniasis is considered a neglected tropical disease, with most cases occurring among populations with low socioeconomic status. The disease manifests in three main clinical forms: visceral leishmaniasis (VL), mucocutaneous leishmaniasis (MCL), and cutaneous leishmaniasis (CL) (Alvar et al., 2012). Several factors contribute to the global spread of the disease, including limited access to healthcare among impoverished communities, poor nutrition, and inadequate sanitation (Boelaert et al., 2009; Grifferty et al., 2021; Herrero et al., 2009; Pigott et al., 2014). The vectors responsible for transmitting New World species are sandflies of the genus *Lutzomyia*. These parasites primarily infect animals, with humans serving as secondary hosts (Basano and Camargo, 2004; Lewis, 1974).

In Brazil, the disease disproportionately affects individuals with low education levels, economic vulnerability, and poor employment conditions, primarily in rural areas (Oliveira et al., 2016; Melo et al., 2020; Vasconcelos et al., 2017). The consequences of CL are both physical and psychological, impacting not only the health of patients but also the economy of the affected regions. CL presents high morbidity, which can interfere with the patient’s physical condition and work productivity, leading to significant economic losses (Bezerra et al., 2018). Among the various species responsible for the disease, *L. amazonensis* is particularly noteworthy due to its high prevalence in the New World and its association with CL (Saidi et al., 2023). This form manifests as chronic skin lesions, which can lead to severe disfigurement and social stigma, underscoring the urgent need for effective therapeutic interventions (Burza et al., 2018; Alemayehu and Alemayehu, 2017; Bennis et al., 2017).

*Leishmania amazonensis* causes severe cutaneous lesions in mice and can induce the immune system to produce a mixed cytokine profile (Pereira and Alves, 2008). The cytokines secreted in response to this species play a crucial role in the parasite’s lifecycle, facilitating tissue invasion, nutrient acquisition, and evasion of the host immune response. Although several mechanisms have been proposed, the anergic nature of *L. amazonensis* remains unclear (Real et al., 2013; Rêgo et al., 2022).

CL cure depends on the type of immune response, particularly one mediated by T helper 1 (Th1) cells (Taraghian et al., 2021). The Th1 response is characterized by high levels of cytokines such as interleukin-12 (IL-12), which promotes the differentiation of T Helper 0 (Th0) cells into Th1 cells; interleukin-1 (IL-1); and interferon-gamma (INF-γ), which stimulates the production of superoxides (O^−2^) and NO, key components for parasite elimination by phagocytes. Tumor necrosis factor-alpha (TNF-α) further enhances the production of superoxides (Von Stebut et al., 2003; Mansueto et al., 2007). In contrast, patients who do not achieve clinical cure typically exhibit a dominant T helper 2 (Th2)-mediated response, with elevated expression of interleukin-10 (IL-10), which promotes an anti-inflammatory effect that hinders effective parasite clearance (Taraghian et al., 2021; Sacks and Noben-Trauth, 2002).

This species has been identified in patients with diverse clinical forms of the disease, including localized cutaneous leishmaniasis (LCL), anergic diffuse cutaneous leishmaniasis (ADCL), MCL, and canine visceral leishmaniasis (CVL), particularly in South American countries and mainly Brazil (Rêgo et al., 2022; Silveira et al., 2004). Among these, ADCL is the most challenging to treat with conventional drugs (Taraghian et al., 2021; Sacks and Noben-Trauth, 2002). It is characterized by numerous nodules and lesions covering large body areas (Silveira et al., 2004; Silveira et al., 2009). In ADCL patients, there is elevated expression of interleukin-4 (IL-4) and IL-10, along with low expression of IFN-γ, reflecting the anergic immune response typical of this condition (Bomfim et al., 1996). In fact, *Leishmania infantum* and *L. amazonensis* can cause the visceral form in dogs; in addition, *L. amazonensis* exhibits natural resistance to antileishmanial drugs, which may contribute to therapeutic failure (Rêgo et al., 2022; Ferreira et al., 2024).

Few medications are available to treat leishmaniasis; among them, pentavalent antimony (SbV) compounds have remained the first-line treatment for several decades in endemic areas such as Brazil, despite their low efficacy rates (Ferreira et al., 2024; Uliana et al., 2018). In addition to the ineffective immune response associated with ADCL caused by *L. amazonensis*, first-line drugs like meglumine antimoniate and second-line treatments such as amphotericin B and liposomal amphotericin have proven ineffective for this clinical form (Costa et al., 2009).

Current treatment options for leishmaniasis predominantly rely on chemotherapeutic agents, such as SbV compounds, amphotericin B, and miltefosine (Fischer et al., 2024). However, serious side effects are associated with many standard formulations, including meglumine antimoniate (Glucantime®) and sodium stibogluconate (Pentostam®), as well as alternative medications like liposomal amphotericin B (AmBisome®), pentamidine, allopurinol, paromomycin, and azole derivatives (Bamorovat et al., 2024; Firooz et al., 2021). These treatments face several challenges, such as high toxicity, variable efficacy, and the emergence of drug resistance (Domínguez-Carmona et al., 2010; Tambe et al., 2024). Their invasive nature and significant side effects also hinder patient compliance and overall treatment success. Given these limitations, exploring alternative therapeutic strategies is imperative.

Natural compounds have attracted considerable interest in recent years due to their potential efficacy and lower toxicity profiles (Wong et al., 2014; Deethamvali et al., 2024; Orosco et al., 2024). Much of the knowledge regarding the therapeutic use of plants is passed down orally through folklore, particularly in the Brazilian Amazon Forest. Plants represent a valuable resource for pharmacological research against parasites, given the long-standing coexistence of herbal treatments, humans, and parasitic diseases (Id et al., 2020; Deethamvali et al., 2024; Orosco et al., 2024). Moreover, natural products offer exceptional structural diversity compared to conventional combinatorial chemistry, facilitating the discovery of novel low molecular-weight lead compounds (Orosco et al., 2024; Cragg and Newman, 2005). It is estimated that nearly 90% of all plant species have yet to be investigated for their potential as antileishmanial agents (Getti et al., 2009). Key factors driving the search for new drugs include limited access to chemotherapy for parasitic infections, the high cost of treatment in endemic regions, increased travel to these areas, and the resulting need for effective prophylaxis, as well as the growing resistance to conventional drugs (Fernández et al., 2024; Anthony et al., 2005).

Numerous plant-derived compounds and secondary metabolites, including terpenoids, flavonoids, alkaloids, and essential oils, have shown antileishmanial activity in both *in vitro* and *in vivo* studies (Orosco et al., 2024; Fernández et al., 2024; Haq et al., 2021; Azim et al., 2021). For example, berberine, a plant-derived alkaloid, has demonstrated significant leishmanicidal effects by inhibiting parasite growth and inducing apoptosis (Hassan et al., 2022; Orosco et al., 2024). Curcumin, a compound found in turmeric, possesses strong immunomodulatory and anti-inflammatory properties that enhance its antileishmanial efficacy (Hassan et al., 2022; Deethamvali et al., 2024; Clemente et al., 2024). Additionally, essential oils from *Artemisia annua* and *Melaleuca alternifolia* have shown notable antileishmanial activity (Hassan et al., 2022; Deethamvali et al., 2024; Orosco et al., 2024).

Flavonoids are a class of natural polyphenolic compounds and secondary metabolites produced via the phenylpropanoid pathway in a wide range of plant species (Liga and Paul, 2023; Dias et al., 2021). They are classified into six major categories: (i) flavanones, (ii) flavones, (iii) isoflavones, (iv) flavonols, (v) flavanols, and (VI) anthocyanins (Pietta et al., 2003). This group of natural compounds has attracted significant research interest due to their diverse biological activities and therapeutic potential. For example, quercetin, a flavonol found in many fruits and vegetables, has been shown to inhibit parasite proliferation, stimulate the production of reactive oxygen species (ROS) that induce cell death in *L. amazonensis*, and modulate host immune responses (Fernández et al., 2024; Haq et al., 2021; Azim et al., 2021).

Naringenin, a citrus flavanone found abundantly in citrus fruits, is a glycosylated flavonoid composed of the flavanone naringenin and the disaccharide neohesperidoside. It is primarily derived from yellowish dihydroflavonoids extracted from the dried peel of Rutaceae plants and grapefruits (Joshi et al., 2018; Olas, 2020; Peng et al., 2024). Naringenin has potent anti-inflammatory properties, making it effective in relieving and treating a wide range of inflammatory conditions, including airway inflammation (Peng et al., 2024). It exhibits neuroprotective and renal effects, in addition to therapeutic potential in the prevention and management of metabolic syndrome and cardiovascular diseases (Joshi et al., 2018; Peng et al., 2024). Its antioxidant and anti-inflammatory properties suggest potential applications in treating protozoan infections. Although further studies are needed to assess its efficacy against pathogens, naringenin may serve as a complementary agent alongside conventional treatments for leishmaniasis.

Despite growing interest in natural compounds for treating leishmaniasis, comprehensive evaluations of their efficacy, mechanisms of action, and potential as viable therapeutic agents are still limited. To address this gap, we conducted a systematic review of the literature focusing on the use of flavonoids against *L. amazonensis*. By advancing our understanding of the antileishmanial potential of these natural compounds, we hope to contribute to the development of safer, more effective, and more accessible treatment options for this disease.

## 2 Methods

### 2.1 Study identification and selection

A systematic search was conducted in the MEDLINE (via PubMed) and Google Scholar databases in 2024 to identify relevant studies on natural compounds used in the treatment of *L. amazonensis*. As this is a systematic review, ethical approval and informed consent were not applicable. All articles that matched the predefined keywords and aligned with the study objective were considered for inclusion. This review adhered to the methodological guidelines outlined in the PRISMA Statement (Moher et al., 2009).

The search encompassed studies published between January 1994 and June 2024 and focused on natural compounds with potential therapeutic effects against *L. amazonensis*. Table 1 details the search strategy, including indexed terms and inclusion and exclusion criteria. Additionally, references cited in the selected publications were screened for further relevant studies.

**TABLE 1:** Search strategy and inclusion/exclusion criteria applied in the systematic review of natural compounds used in the treatment of *Leishmania amazonensis* infection.

Index terms	
Pubmed (*Leishmania amazonensis*) AND (((biological products) OR (medicinal plant) OR (natural compounds))) OR (Chemical treatment) OR (Flavonoid) OR (Cutaneous diffuse) OR (Synergism)	Google Scholar (*Leishmania amazonensis*) (biological products or medicinal plants or natural compounds)
Applied Criteria	
Inclusion Studies evaluating natural compounds for antileishmanial activity. Studies assessing the synergistic effects of natural compounds combined with commercial drugs	Exclusion Studies involving other *Leishmania* species Studies testing synthetic chemical compounds Dissertations, theses, review articles, book chapters, and letters to the editor

### 2.2 Methodological quality assessment

The methodological quality of the studies included in this review was assessed independently by two reviewers (Vinícius Lessa and Guilherme Drescher – VL and GD, respectively). The evaluation focused specifically on studies that tested flavonoids against *L. amazonensis*, with inclusion restricted to those using *in vitro* assays.

For each article, we examined the type of solvent used for the extraction and isolation of the compounds, as well as the methods used to characterize the flavonoids. We also identified the type of diluents employed to dissolve the flavonoids for testing purposes. Particular attention was paid to whether the studies used colorimetric assays to evaluate antileishmanial activity and assessed the cytotoxic concentration 50 (CC_50_) in mammalian cells.

Furthermore, we verified whether the studies reported the half-maximal inhibitory concentration (IC_50_) against promastigote and amastigote forms and whether CC_50_ values were also determined. In all selected articles, we investigated whether the selectivity index (SI) was calculated for either isolated flavonoids or mixtures present in the solvent extracts from biological material.

Additionally, we evaluated whether the studies conducted synergism assays in promastigote and amastigote forms to determine the type of interaction between flavonoids, as well as between flavonoids and commercial drugs. We also extracted information regarding any proposed mechanisms of action against both parasite forms, and if any *in silico* assays were performed. Finally, we checked which types of experimental controls were used in each study.

If necessary, additional information was requested from the authors of the included studies. Any discrepancies in data extraction were resolved through group discussion, with the assistance of a third evaluator.

### 2.3 Activity against the parasite and cytotoxicity assays

For all studies characterizing flavonoids, we evaluated their antileishmanial activity based on their ability to inhibit parasite growth. An extract or compound was considered active if it exhibited an IC_50_ value of ≤ 10 μg/mL against promastigote or amastigote forms. Moderate activity was defined as an IC_50_ value between > 10 μg/mL and < 50 μg/mL, while weak activity was assigned to those with IC_50_ values between ≥ 50 µg/mL and 100 μg/mL. Only the IC_50_ values of the characterized flavonoids were included in this review.

To assess treatment efficacy, we used the SI, considering values of SI ≥ 10 as indicative of high therapeutic potential, since such values suggest greater selectivity for the parasite over host cells (Hassan et al., 2022).

To determine the nature of the interaction between natural compounds, we used the fractional inhibitory concentration index (FICI). A FICI ≥ 0.5 indicates a synergistic effect, values between 0.5 > and ≤ 4 indicate an additive effect, and values > 4 denote an antagonistic effect (Seifert and Croft, 2006).

## 3 Results

The initial search retrieved 208 articles from PubMed and 1,137 from Google Scholar databases. Of these, 579 titles or abstracts were initially selected for evaluation based on the search strategy. After removing 26 duplicates, 506 records remained for screening.

**FIGURE 1:**
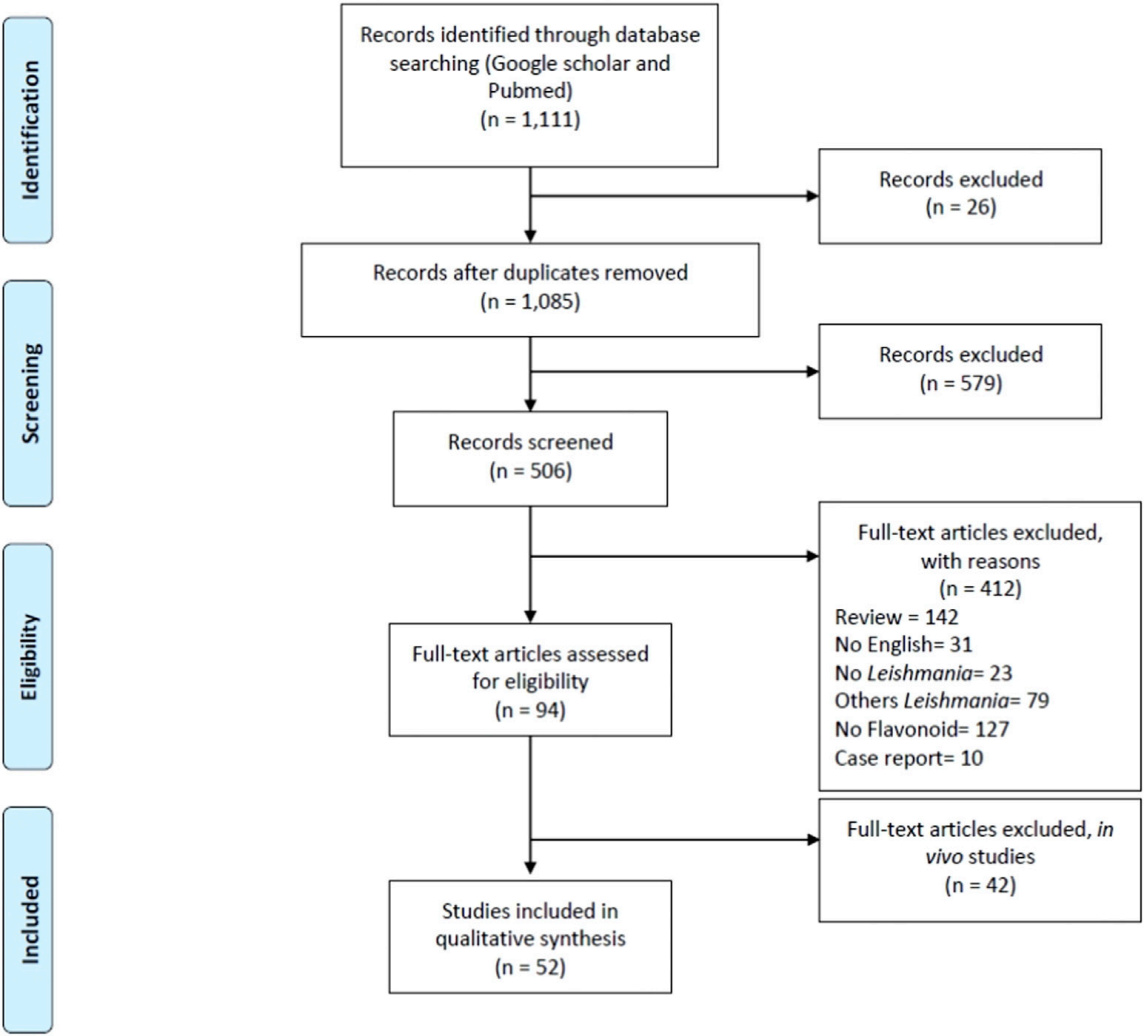
PRISMA flow diagram (Moher et al., 2009).

Out of these, 142 were excluded as review articles, and 31 were excluded for being published in languages other than English. An additional 23 studies were excluded for not addressing *Leishmania*, and 79 were excluded for focusing on *Leishmania* species other than *L. amazonensis*. A further 83 articles were excluded for not involving flavonoid compounds, and 10 papers were removed because they were case reports. Ninety-four full-text articles were assessed for eligibility, of which 42 were excluded for focusing on *in vivo* studies. Ultimately, 52 studies met the inclusion criteria and were included in the qualitative synthesis (Figure 1).

### 3.1 Flavonoid extraction

The extraction of flavonoids from plant matrices highly depends on the polarity of both the target compounds and the solvents used. In this review, the most frequently employed solvents were organic solvents such as ethanol (EtOH), methanol (MeOH), and ethyl acetate (EtOAc). EtOH and MeOH were primarily used for more polar flavonoids, while less polar flavonoids were extracted with solvents such as acetone (Ac), chloroform (Chl), dichloromethane (DCM), diethyl ether (Et2O), and hexane (Hx) to optimize yield and purity. In some studies, more than one solvent was used; for example, Delgado-Altamirano et al. (2017) employed DCM alone, an aqueous solvent, and a mixture of DCM with methanol to extract flavonoids.

### 3.2 Diluents for *in vitro* assays

For *in vitro* bioactivity and cytotoxicity assays, dimethyl sulfoxide (DMSO) was the predominant diluent, chosen for its superior solubilizing capacity and compatibility with a wide range of flavonoid structures. Some protocols also employed ethanol, methanol, and other solvents as secondary diluents. The choice of diluent was closely aligned with the solubility profile of the flavonoid under investigation and the requirements of the bioassay system. Deuterated chloroform (CDCl_3_) was used as a diluent in the work by Santos et al. (2019), along with DMSO.

### 3.3 Flavonoid characterization methods

Flavonoid content was determined using UV-Vis spectrophotometry through aluminum chloride complexation, exploiting the distinct absorption maxima of flavones and flavonols. In this review, we considered both simpler methods for flavonoid identification, such as fluorescence spectroscopy, and advanced techniques like Ultra-High-Performance Liquid Chromatography coupled with High-Resolution Mass Spectrometry (UHPLC-HRMS). Santos et al. (2019) employed nuclear magnetic resonance (NMR) spectroscopy, which provided detailed insights into molecular weight, fragmentation patterns, and structural motifs.

### 3.4 Mechanism of action studies on promastigote and amastigote forms

Among the 52 studies included in this systematic review on the effects of flavonoids against *L. amazonensis*, only 11 (21%) included investigations into the mechanism of action against the parasite’s promastigote form. Within these studies, at least four different experimental approaches were employed, with transmission electron microscopy (TEM) being the most frequently used technique.

In addition, 16 (31%) of the 52 studies included investigations on the mechanism of action against the amastigote form. Within these 16 studies, at least eight different experimental approaches were used, with nitric oxide (NO) evaluation as the most frequently applied method.

### 3.5 *In silico* assays on amastigote forms

Among the 52 studies reviewed, only 10 (about 19%) explored the mechanism of action against the amastigote form using *in silico* assays. These studies employed at least five different *in silico* methodologies to investigate potential mechanisms. The most commonly used tools included docking with AutoDock Vina® for analyzing and predicting molecular interactions, Swiss ADME® for predicting physicochemical properties, and pkCSM for predicting toxicity. Molecular docking was the most frequently applied approach, featured in four of the studies.

### 3.6 Quality assessment of included studies

Three articles fulfilled at least 13 of the 18 quality assessment criteria (70%) listed in Supplementary Tables S1, S2 and were considered the most complete studies. Twenty-six articles met between nine and 12 criteria (50%–65%) and were classified as regular studies. The remaining 23 articles satisfied eight or fewer criteria (45%–23%), suggesting lower methodological rigor (see Supplementary Table S1).

Of the 52 included studies, antileishmanial activity of specific plant-derived fractions was assessed in 38 (73%). Moreover, the effects of isolated and characterized flavonoids against *L. amazonensis* were explored in 41 studies (78%).

### 3.7 Activity against the parasite and cytotoxicity assays

Articles were included if they demonstrated a significant antileishmanial effect and provided characterization of the flavonoids tested, including data obtained from colorimetric and plate reader assays. IC_50_ and CC_50_ values from *in vitro* experiments of these studies are summarized in Supplementary Tables S3, S4.

A total of 69 flavonoids were identified across the reviewed studies and evaluated for IC_50_ values against both amastigote (Supplementary Chart S1) and promastigote forms (Supplementary Chart S2) of *L. amazonensis*, as well as CC_50_ values in cytotoxicity assays, according to the criteria described in Section 2.3.

Among the assays targeting amastigotes, 30 showed high activity (IC_50_ ≤ 10 μg/mL), six demonstrated moderate activity (10 μg/mL < IC_50_ < 50 μg/mL), and none were classified as weak (Supplementary Table S3). For promastigote forms, high activity was observed in 32 assays, moderate activity in 16, and weak activity in two (Supplementary Table S3).

The most active flavonoid against promastigotes was (+/−) fukugiside, with an IC_50_ of 0.0320 μg/mL. Against amastigotes, the greatest activity was observed for morelloflavone (IC_50_ = 0.161 μg/mL). In contrast, naringenin was the least active compound, with an IC_50_ of 59.87 μg/mL reported only for promastigotes.

Regarding cytotoxicity, the highest SI was 34.8 for carajurin against amastigotes and 32.4 against promastigotes. The lowest SI values were 1.1 for amastigotes and 0.41 for promastigotes, both associated with luteolin.

## 4 Discussion

This review synthesizes key methodological approaches used *in vitro* research and discusses their implications for developing alternative therapeutic strategies against *L. amazonensis*. The analysis focused on the biological effects of flavonoids derived from plants and propolis on *L. amazonensis*, a protozoan parasite responsible for a form of CL prevalent in tropical regions.

Numerous natural compounds have been isolated from different plant parts traditionally used in folk medicine to treat leishmaniasis (Fabri et al., 2009; Araújo et al., 2019; Ferreira et al., 2021; Fróes et al., 2023), underscoring the relevance of fractionation techniques in identifying bioactive constituents and assessing their therapeutic potential against *L. amazonensis*. More than 30 compounds with activity against *L. amazonensis* were identified, reflecting the chemical diversity of the isolated substances. In addition to plant-derived flavonoids, phenolic compounds isolated from propolis were also investigated (Cuesta-Ru et al., 2017; Cavalcante et al., 2021; Dutra et al., 2023).

The key solvents used in the reviewed studies were Hx, EtOAc, and MeOH, highlighting the importance of solvent selection in extracting plant-derived bioactive compounds (Ferreira et al., 2021; Fróes et al., 2023; Dutra et al., 2023; Salvador et al., 2009; Silva-Silva et al., 2021). The choice of solvent influences both the chemical profile of the resulting extracts and their solubility and bioavailability in downstream assays.

Over 70% of the reviewed studies employed DMSO as the primary solvent for diluting bioactive compounds prior to testing (Salvador et al., 2002; Clavin et al., 2016; Mai et al., 2015; de Araújo et al., 2024). Notably, about 20% did not use MTT (3-[4,5-dimethylthiazol-2-yl]-2,5-diphenyl tetrazolium bromide) or resazurin (7-hydroxy-3H-phenoxazin-3-one-10-oxide) as their primary colorimetric methods to evaluate antileishmanial activity (Mai et al., 2015; Correia et al., 2016; Araújo et al., 2022; Lessa et al., 2024). Alternative methods included adenosine triphosphate (ATP) quantification, flow cytometry, and direct microscopic counting, employed in a total of 12 papers.

*In vitro* assays, these compounds demonstrated varying levels of efficacy (Supplementary Table S3), with several showing promising antileishmanial activity while maintaining low cytotoxicity toward mammalian cells. Gontijo et al. (2012) identified morelloflavone-4‴O-β-D-glycosyl, isolated from *Garcinia brasiliensis*, as the most active compound against both amastigote and promastigote forms, with an IC_50_ of 0.0234 μg/mL. Salvador et al. (2009) reported pinostrobin as the most active compound against amastigotes, with an IC_50_ of 0.0838 μg/mL. Conversely, naringenin was the least effective compound against promastigotes, with an IC_50_ of 59.87 μg/mL. Sakuranetin showed moderate activity against amastigotes (IC_50_ = 51.89 μg/mL; 39.31 ± 69.98), based on the classification by Hassan et al. (2022).

Another important parameter is SI, which considers both efficacy and cytotoxicity (Supplementary Table S4). Among the flavonoids reviewed, luteolin showed the lowest SI value (SI = 0.679), while carajurin had the highest (SI = 34.8). Treatment efficiency can be assessed based on SI values, as an SI value greater than 10 indicates a compound with greater selectivity and promising potential for further investigation (Peña-Morán et al., 2016). Despite the high activity values observed for morelloflavone-4‴O-β-D-glycosyl and pinostrobin, and the lower activity observed for naringenin, the SI alone was not used to support a more in-depth analysis of these compounds. However, determining the CC_50_ is essential for calculating SI values.

The relatively limited number of *in silico* investigations reveals an important gap in the current research, given the valuable insights these techniques provide into molecular interactions and potential targets. Docking studies, in particular, have proven instrumental in predicting binding affinities and interaction modes between flavonoids and key *Leishmania* enzymes, such as arginase and trypanothione reductase. These computational results complement *in vitro* findings and help elucidate possible mechanisms underlying antileishmanial activity (Fróes et al., 2023; Dutra et al., 2023; Silva-Silva et al., 2021; Santos et al., 2019). The diversity of *in silico* methodologies applied also reflects the complexity of flavonoid action and highlights the need for multifaceted computational approaches to fully explore their pharmacological potential (Rizk et al., 2021; Santos et al., 2019; Gervazoni et al., 2018).

Although naringenin showed the lowest IC_50_ value for promastigote forms compared to other flavonoids analyzed in these studies—and even when compared to commercial drugs like miltefosine—this flavonoid also demonstrated potent *in vitro* activity against other *Leishmania* species, such as *L. donovani*. It activates CD4^+^ and CD8^+^ T cells, as well as Th1-type cytokines, which enhance the host immune response against the parasite. Moreover, its lower *in vitro* toxicity when used in monotherapy suggests it may help minimize the side effects typically associated with commercial drugs (Kaur et al., 2018).

Another aspect to emphasize is the potential of drug combinations to improve treatment outcomes. Naringenin exhibited an additive effect with miltefosine against promastigote forms of *L. amazonensis*, allowing the dose of this compound to be reduced by approximately half while maintaining the same efficacy observed when the drug was used alone *in vitro* assays (Lessa et al., 2024).

Among the 52 studies analyzed in this review, only two presented FICI assays for promastigote forms and two for amastigote forms (Supplementary Table S2). This is a particularly relevant topic, as calculating FICI enables the investigation of the interactions between flavonoids and drugs and opens up the possibility for optimizing leishmaniasis treatment.

## 5 Conclusion

This review aimed to improve access to information by updating and summarizing recent research on flavonoid compounds against *L. amazonensis*. Flavonoids derived from natural sources, including plants and propolis, have demonstrated a wide range of activities against different forms of this species, with some exhibiting high levels of efficacy that could represent promising leads for the development of innovative, affordable drugs.

Most of the studies reviewed focused on the promastigote form of the *L. amazonensis*. *In vitro* assays remain crucial for screening extracts and isolated flavonoids, as well as for investigating their cellular and molecular mechanisms of action. Studying natural compounds, particularly flavonoids, remains highly relevant for advancing knowledge on *L. amazonensis* infection and for guiding the development of more effective therapeutic strategies.

## Data Availability

The original contributions presented in the study are included in the article/Supplementary Material, further inquiries can be directed to the corresponding author.
