# Supplementary material for: Flavonoids in the treatment of Leishmania amazonensis: a review of efficacy and mechanisms
Source: Front Pharmacol. 2025 Aug 7;16:1642005. doi: 10.3389/fphar.2025.1642005 (PMC12367659; doi:10.3389/fphar.2025.1642005)
Supplement: Supplementary file 1 [file Supplementaryfile1.docx]

Supplementary Chart – S1: Chemical structure of the most active flavonoid against amastigote forms

|  | Characterized flavonoid | Chemical Structure | Pubchem CID |
| --- | --- | --- | --- |
| Dutra et al 2023  Silva et al 2019 | Atalantoflavone (Erythrina sigmoidea) | 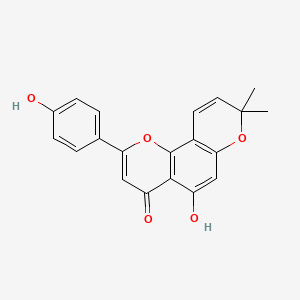 | CID 14162621 |
| Silva et al 2021 | Carajurin | 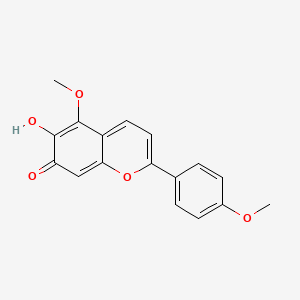 | CID 9948177 |
| Rizk et al 2021  Silva et al 2022  Rizk et al 2022  Rizk et al 2014 | Amentoflavone | 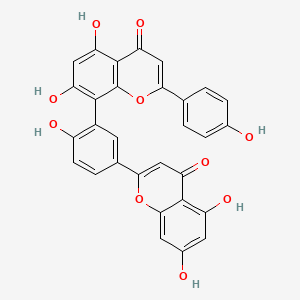 | CID 5281600 |
| Morais et al 2020 | Hemileiocarpin | 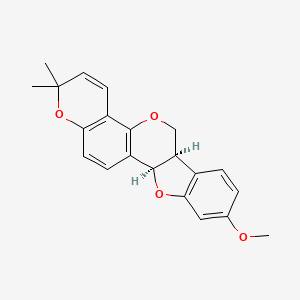 | CID 70995758 |
| Silva et al 2019 | Abyssinone IV (Erythrina sigmoidea) | 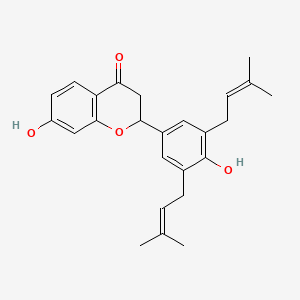 | CID 4063835 |
| Rocha et al 2019  Dal Picolo et al 2014 | Brachydin B (dimeric flavonoid) | 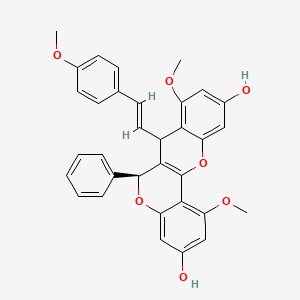 | SID 275575602 |
|  | Brachydin C (dimeric flavonoid) | 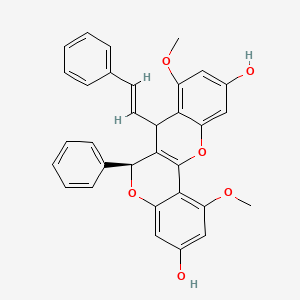 | SID 275575601 |
| Emiliano_&_Almeida-Amaral 2018  Fonseca-Silva et al 2016  Fonseca-Silva et al 2015  Silva et al 2021 | Apigenin | 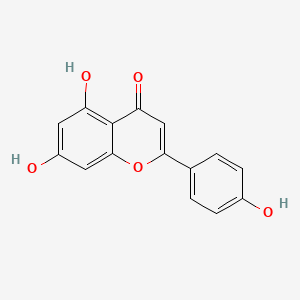 | CID 5280443 |
| Rizk et al 2014 | Robustaflavone | 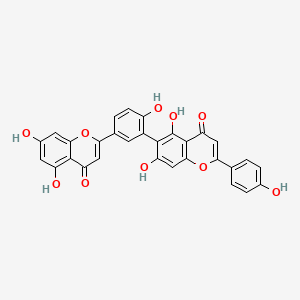 | CID 5281694 |
| Assolini et al 2020 | 4-nitrochalcone (4NC) (comercial) | 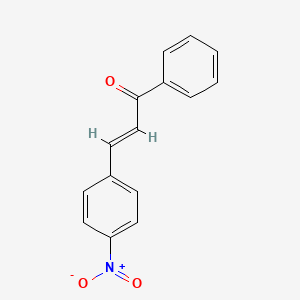 | CID 5377323 |
| Manjolin et al 2013 |  |  |  |
|  | Quercitrin | 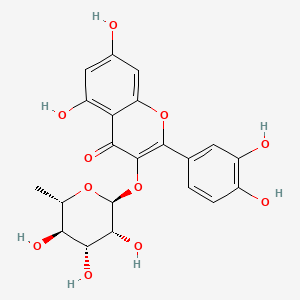 | CID 5280459 |
|  | 7,8-dihydroxyflavone | 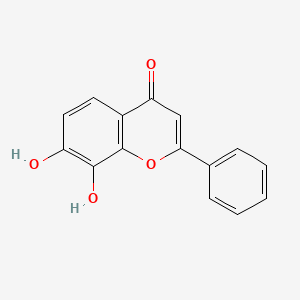 | CID 1880 |
|  | Fisetin | 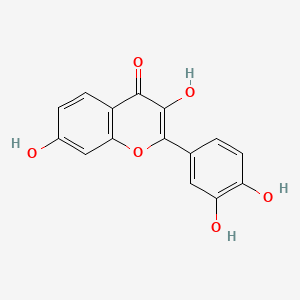 | CID 5281614 |
|  | Quercetin | 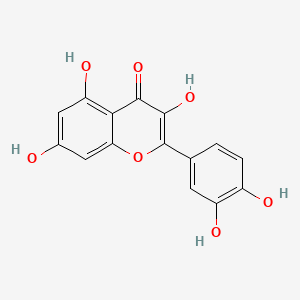 | CID 5280343 |
|  | Luteolin | 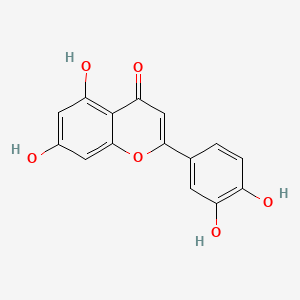 | CID 5280445 |
| Salvador et al 2009 | Pinostrobin | 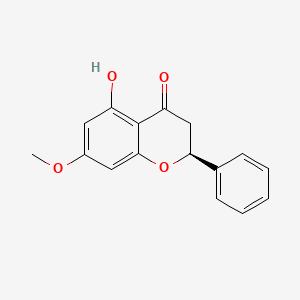 | CID 73201 |
|  | Tectochrysin | 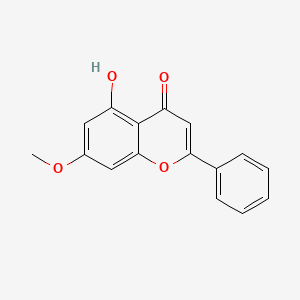 | CID 5281954 |
|  | Galangin 3-methyl ether | 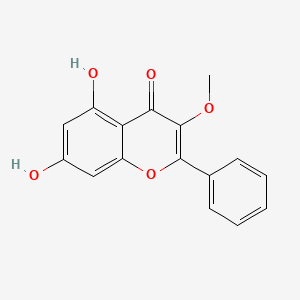 | CID 5281946 |
