# Supplementary material for: Flavonoids in the treatment of Leishmania amazonensis: a review of efficacy and mechanisms
Source: Front Pharmacol. 2025 Aug 7;16:1642005. doi: 10.3389/fphar.2025.1642005 (PMC12367659; doi:10.3389/fphar.2025.1642005)
Supplement: Supplementary file 2 [file Table1.docx]

Supplementary table – S1. List of papers with each assessment of quality results by selected criteria.

|  | Solvent | Characterization Methods | Characterized Flavonoid | Diluent | Flavonoid Quantification | Colorimetric Methods for Citoxicity and Antileishmanial Assay | IC_50_ Amastigote | IC_50_ Promastigote | CC_50_ |
| --- | --- | --- | --- | --- | --- | --- | --- | --- | --- |
| Araújo et al 2024 | Yes |  |  | Yes | Yes | Yes | Yes | Yes | Yes |
| Dutra et al 2023 | Yes | Yes | Yes |  | Yes | Yes | Yes | Yes | Yes |
| Pacheco et al 2023 | Yes | Yes | Yes |  |  | Yes | Yes | Yes | Yes |
| Fróes et al 2023 | Yes | Yes | Yes |  |  | Yes |  | Yes | Yes |
| Araújo et al 2022 |  |  | Yes | Yes |  | Yes |  | Yes | Yes |
| Silva et al 2022 | Yes | Yes | Yes |  |  | Yes |  | Yes | Yes |
| Bezerra et al 2021 | Yes | Yes | Yes | Yes |  | Yes |  | Yes | Yes |
| Rizk et al 2022 | Yes |  | Yes | Yes |  | Yes |  | Yes |  |
| Silva et al 2021 | Yes | Yes | Yes |  | Yes | Yes | Yes | Yes | Yes |
| Silva et al 2021 | Yes | Yes | Yes |  | Yes | Yes | Yes | Yes | Yes |
| Cavalcante et al 2021 | Yes | Yes | Yes | Yes |  | Yes | Yes | Yes | Yes |
| Rizk et al 2021 |  |  | Yes | Yes |  | Yes | Yes |  | Yes |
| Ferreira et al 2021 | Yes | Yes | Yes |  |  | Yes |  | Yes | Yes |
| Morais et al 2020 | Yes | Yes | Yes |  |  | Yes | Yes | Yes | Yes |
| Silva et al 2019 | Yes | Yes | Yes | Yes | Yes | Yes | Yes |  | Yes |
| Santos et al 2019 | Yes | Yes |  | Yes |  | Yes | Yes | Yes | Yes |
| Rocha et al 2019 |  | Yes | Yes | Yes | Yes | Yes | Yes | Yes | Yes |
| Emiliano & Almeida-Amaral 2018 |  |  | Yes | Yes | Yes | Yes | Yes |  |  |
| Almeida-Souza et al 2018 | Yes | Yes |  | Yes |  | Yes | Yes |  |  |
| Fadel et al 2018 | Yes |  |  | Yes | Yes | Yes | Yes |  | Yes |
| Delgado-Altamirano et al 2017 | Yes | Yes |  | Yes |  | Yes | Yes | Yes | Yes |
| Cuesta-Rubio et al 2017 | Yes | Yes |  | Yes |  | Yes | Yes | Yes | Yes |
| Correia et al 2016 | Yes |  |  | Yes | Yes | Yes |  | Yes | Yes |
| Duarte et al 2016 | Yes | Yes |  |  | Yes | Yes | Yes | Yes | Yes |
| Fonseca-Silva et al 2016 |  |  | Yes | Yes | Yes |  | Yes |  |  |
| Fonseca-Silva et al 2015 |  |  | Yes | Yes | Yes |  |  | Yes |  |
| Mai et al 2015 | Yes | Yes | Yes | Yes | Yes | Yes |  | Yes |  |
| Rizk et al 2014 | Yes | Yes | Yes | Yes |  |  | Yes |  |  |
| Assolini et al 2020 |  |  | Yes | Yes |  | Yes | Yes | Yes | Yes |
| Zeouk et al 2020 | Yes | Yes | Yes | Yes |  |  | Yes | Yes | Yes |
| Oliveira et al 2021 | Yes | Yes | Yes |  |  |  | Yes | Yes |  |
| Fadel et al 2019 | Yes |  |  | Yes | Yes |  | Yes | Yes | Yes |
| Araújo et al 2019 | Yes | Yes | Yes | Yes |  | Yes | Yes | Yes | Yes |
| Cabanillas et al 2014 | Yes | Yes | Yes | Yes |  | Yes | Yes | Yes | Yes |
| Dal Picolo et al 2014 | Yes | Yes | Yes | Yes |  | Yes | Yes | Yes | Yes |
| Ribeiro et al 2014 | Yes | Yes | Yes | Yes |  | Yes |  | Yes | Yes |
| Wong el al 2014 |  | Yes |  | Yes |  | Yes | Yes | Yes | Yes |
| Lage et al 2013 | Yes | Yes | Yes | Yes |  | Yes |  | Yes | Yes |
| Manjolin et al 2013 |  | Yes | Yes | Yes |  |  | Yes |  |  |
| Gervazoni et al 2018 |  |  | Yes | Yes |  |  | Yes | Yes | Yes |
| Fabri et al 2009 | Yes | Yes |  | Yes | Yes | Yes |  | Yes | Yes |
| Silva et al 2011 |  |  | Yes | Yes |  | Yes |  | Yes |  |
| Gontijo et al 2012 | Yes | Yes | Yes | Yes |  | Yes | Yes | Yes | Yes |
| Grecco et al 2012 | Yes | Yes | Yes | Yes |  | Yes | Yes | Yes | Yes |
| Machado et al 2007 | Yes | Yes | Yes | Yes |  |  |  | Yes |  |
| Pereira et al 2011 | Yes | Yes | Yes | Yes | Yes |  | Yes |  |  |
| Salvador et al 2009 | Yes | Yes | Yes | Yes |  | Yes | Yes |  |  |
| Taled-Contini et al 2004 | Yes | Yes | Yes | Yes | Yes | Yes |  | Yes |  |
| Lessa et al 2024 |  |  | Yes | Yes | Yes | Yes |  | Yes |  |
| Inacio et al 2013 |  |  | Yes | Yes | Yes |  | Yes |  |  |
| Salvador et al 2002 | Yes | Yes | Yes | Yes | Yes | Yes |  |  |  |
| Clavin et al 2007 | Yes | Yes | Yes | Yes |  |  |  |  |  |
