# Supplementary material for: Flavonoids in the treatment of Leishmania amazonensis: a review of efficacy and mechanisms
Source: Front Pharmacol. 2025 Aug 7;16:1642005. doi: 10.3389/fphar.2025.1642005 (PMC12367659; doi:10.3389/fphar.2025.1642005)
Supplement: Supplementary file 3 [file Supplementaryfile3.docx]

List references:

Almeida-Souza F, De Oliveira AER, Abreu-Silva AL, Da Silva Calabrese K. In vitro activity of Morinda citrifolia Linn. fruit juice against the axenic amastigote form of Leishmania amazonensis and its hydrogen peroxide induction capacity in BALB/c peritoneal macrophages. Vol. 11, BMC Research Notes. 2018.

Assolini JP, da Silva TP, da Silva Bortoleti BT, Gonçalves MD, Tomiotto-Pellissier F, Sahd CS, et al. 4-nitrochalcone exerts leishmanicidal effect on L. amazonensis promastigotes and intracellular amastigotes, and the 4-nitrochalcone encapsulation in beeswax copaiba oil nanoparticles reduces macrophages cytotoxicity. Eur J Pharmacol. 2020;884(March).

Bezerra ÉA, Alves MM de M, Lima SKR, Pinheiro EEA, Amorim LV, Lima Neto J de S, et al. Biflavones from platonia insignis mart. Flowers promote in vitro antileishmanial and immunomodulatory effects against internalized amastigote forms of leishmania amazonensis. Pathogens. 2021;10(9).

Cabanillas BJ, Le Lamer AC, Olagnier D, Castillo D, Arevalo J, Valadeau C, et al. Leishmanicidal compounds and potent PPARγ activators from Renealmia thyrsoidea (Ruiz & Pav.) Poepp. & Endl. J Ethnopharmacol. 2014;157:149–55.

Da Silva LAL, De Moraes MH, Scotti MT, Scotti L, De Jesus Souza R, Nantchouang Ouete JL, et al. Antiprotozoal investigation of 20 plant metabolites on Trypanosoma cruzi and Leishmania amazonensis amastigotes. Atalantoflavone alters the mitochondrial membrane potential. Parasitology. 2019;146(7):849–56.

Dal Picolo CR, Bezerra MP, Gomes KS, Passero LFD, Laurenti MD, Martins EGA, et al. Antileishmanial activity evaluation of adunchalcone, a new prenylated dihydrochalcone from Piper aduncum L. Fitoterapia [Internet]. 2014;97:28–33. Available from: <http://dx.doi.org/10.1016/j.fitote.2014.05.009>

de Oliveira DP, de Almeida L, Marques MJ, de Carvalho RR, Dias ALT, da Silva GA, et al. Exploring the bioactivity potential of Leonotis nepetifolia: phytochemical composition, antimicrobial and antileishmanial activities of extracts from different anatomical parts. Nat Prod Res [Internet]. 2021;35(18):3120–5. Available from: <https://doi.org/10.1080/14786419.2019.1686367>

Duarte MC, Tavares GSV, Valadares DG, Lage DP, Ribeiro TG, Lage LMR, et al. Antileishmanial activity and mechanism of action from a purified fraction of Zingiber officinalis Roscoe against Leishmania amazonensis. Exp Parasitol [Internet]. 2016;166:21–8. Available from: <http://dx.doi.org/10.1016/j.exppara.2016.03.026>

Emiliano YSS, Almeida-Amaral EE. Efficacy of Apigenin and Miltefosine Combination Therapy against Experimental Cutaneous Leishmaniasis. J Nat Prod. 2018;81(8):1910–3.

Fadel H, Sifaoui I, López-Arencibia A, Reyes-Batlle M, Hajaji S, Chiboub O, et al. Assessment of the antiprotozoal activity of Pulicaria inuloides extracts, an Algerian medicinal plant: leishmanicidal bioguided fractionation. Parasitol Res. 2018;117(2):531–7.

Fadel H, Sifaoui I, López-Arencibia A, Reyes-Batlle M, Jiménez IA, Lorenzo-Morales J, et al. Antioxidant and leishmanicidal evaluation of Pulicaria inuloides root extracts: A bioguided fractionation. Pathogens. 2019;8(4):1–11.

Fonseca-silva F, Inacio JDF, Canto-cavalheiro MM, Almeida-amaral EE. Reactive Oxygen Species Production and Mitochondrial Dysfunction Contribute to Quercetin Induced Death in Leishmania amazonensis. 2011;6(2).

Fonseca-Silva F, Canto-Cavalheiro MM, Menna-Barreto RFS, Almeida-Amaral EE. Effect of Apigenin on Leishmania amazonensis Is Associated with Reactive Oxygen Species Production Followed by Mitochondrial Dysfunction. J Nat Prod. 2015;78(4):880–4.

Fonseca-Silva F, Inacio JDF, Canto-Cavalheiro MM, Menna-Barreto RFS, Almeida-Amaral EE. Oral Efficacy of Apigenin against Cutaneous Leishmaniasis: Involvement of Reactive Oxygen Species and Autophagy as a Mechanism of Action. PLoS Negl Trop Dis. 2016;10(2):1–16.

Grecco S dos S, Reimão JQ, Tempone AG, Sartorelli P, Cunha RLOR, Romoff P, et al. In vitro antileishmanial and antitrypanosomal activities of flavanones from Baccharis retusa DC. (Asteraceae). Exp Parasitol [Internet]. 2012;130(2):141–5. Available from: <http://dx.doi.org/10.1016/j.exppara.2011.11.002>

Inacio JDF, Canto-cavalheiro MM, Almeida-amaral EE, Deane L. In Vitro and in Vivo E ff ects of ( − )-Epigallocatechin 3 ‑ O ‑ gallate on Leishmania amazonensis. 2013;0–3.

Lage PS, De Andrade PHR, Lopes ADS, Chávez Fumagalli MA, Valadares DG, Duarte MC, et al. Strychnos pseudoquina and its purified compounds present an effective in vitro antileishmanial activity. Evidence-based Complement Altern Med. 2013;2013.

Machado GMDC, Leon LL, De Castro SL. Activity of Brazilian and Bulgarian propolis against different species of Leishmania. Mem Inst Oswaldo Cruz. 2007;102(1):73–7.

Manjolin LC, Dos Reis MBG, Do Carmo Maquiaveli C, Santos-Filho OA, Da Silva ER. Dietary flavonoids fisetin, luteolin and their derived compounds inhibit arginase, a central enzyme in Leishmania (Leishmania) amazonensis infection. Food Chem [Internet]. 2013;141(3):2253–62. Available from: <http://dx.doi.org/10.1016/j.foodchem.2013.05.025>

Pacheco JS, Teixeira ÉMGF, Paschoal RG, Torres-Santos EC, Simone SG DE, Silva-López RE DA. Antileishmanial effects of Crotalaria spectabilis Roth aqueous extracts on Leishmania amazonensis. An Acad Bras Cienc. 2023;95:1–23.

Ribeiro TG, Chávez-Fumagalli MA, Valadares DG, Franca JR, Lage PS, Duarte MC, et al. Antileishmanial activity and cytotoxicity of Brazilian plants. Exp Parasitol [Internet]. 2014;143(1):60–8. Available from: <http://dx.doi.org/10.1016/j.exppara.2014.05.004>

Rizk YS, Fischer A, Cunha M de C, Rodrigues PO, Marques MCS, Matos M de FC, et al. In vitro activity of the hydroethanolic extract and biflavonoids isolated from Selaginella sellowii on Leishmania (Leishmania) amazonensis. Mem Inst Oswaldo Cruz. 2014;109(8):1050–6.

Rizk YS, Santos-Pereira S, Gervazoni L, Hardoim D de J, Cardoso F de O, de Souza C da SF, et al. Amentoflavone as an Ally in the Treatment of Cutaneous Leishmaniasis: Analysis of Its Antioxidant/Prooxidant Mechanisms. Front Cell Infect Microbiol. 2021;11(February):1–13.

Rocha VPC, Da Rocha CQ, Queiroz EF, Marcourt L, Vilegas W, Grimaldi GB, et al. Antileishmanial activity of dimeric flavonoids isolated from arrabidaea brachypoda. Molecules. 2019;24(1).
