# Supplementary material for: Flavonoids in the treatment of Leishmania amazonensis: a review of efficacy and mechanisms
Source: Front Pharmacol. 2025 Aug 7;16:1642005. doi: 10.3389/fphar.2025.1642005 (PMC12367659; doi:10.3389/fphar.2025.1642005)
Supplement: Supplementary file 4 [file Table2.docx]

Supplementary table – S2. List with more selected criteria.

|  |  | Selectivity Index (SI) Amastigote | Selectivity Index (SI) Promastigote | Fractional Inhibitory Concentration Index (FICI) Amastigote | Fractional Inhibitory Concentration Index (FICI) Promastigote | Mechanism of Action in Promastigotes | Mechanism of Action in Amastigotes | In silico Assay | Control for Antipromastigotes Assay | Control for Antiamastigote Assay |
| --- | --- | --- | --- | --- | --- | --- | --- | --- | --- | --- |
| Araújo et al 2024 |  | Yes | Yes |  |  |  | Yes |  | Yes | Yes |
| Dutra et al 2023 |  | Yes |  |  |  |  |  | Yes | Yes | Yes |
| Pacheco et al 2023 |  | Yes |  |  |  |  |  |  | Yes | Yes |
| Fróes et al 2023 |  |  | Yes |  | Yes | Yes |  | Yes | Yes |  |
| Araújo et al 2022 |  |  | Yes |  |  | Yes |  |  | Yes |  |
| Silva et al 2022 |  |  | Yes |  |  | Yes |  | Yes | Yes |  |
| Bezerra et al 2021 |  | Yes |  |  |  |  | Yes |  |  | Yes |
| Rizk et al 2022 |  |  |  |  |  | Yes | Yes | Yes | Yes | Yes |
| Silva et al 2021 |  |  |  |  |  | Yes | Yes |  | Yes | Yes |
| Silva et al 2021 |  | Yes |  |  |  |  | Yes | Yes | Yes | Yes |
| Cavalcante et al 2021 |  |  |  |  |  |  |  |  | Yes |  |
| Rizk et al 2021 |  | Yes |  |  |  |  | Yes | Yes |  | Yes |
| Ferreira et al 2021 |  |  | Yes |  |  |  |  |  | Yes |  |
| Morais et al 2020 |  | Yes |  |  |  | Yes |  |  | Yes |  |
| Silva et al 2019 |  | Yes |  |  |  |  |  | Yes | Yes | Yes |
| Santos et al 2019 |  | Yes | Yes |  |  |  |  |  | Yes |  |
| Rocha et al 2019 |  |  |  |  |  |  | Yes |  | Yes | Yes |
| Emiliano & Almeida-Amaral 2018 |  |  |  | Yes |  |  |  |  |  | Yes |
| Almeida-Souza et al 2018 |  | Yes |  |  |  |  | Yes |  |  | Yes |
| Fadel et al 2018 |  | Yes |  |  |  |  |  |  |  |  |
| Delgado-Altamirano et al 2017 |  | Yes | Yes |  |  |  |  |  | Yes | Yes |
| Cuesta-Rubio et al 2017 |  |  | Yes |  |  |  |  |  | Yes | Yes |
| Correia et al 2016 |  |  | Yes |  |  |  |  |  | Yes |  |
| Duarte et al 2016 |  |  |  |  |  |  | Yes |  | Yes | Yes |
| Fonseca-Silva et al 2016 |  |  |  |  |  |  | Yes |  |  | Yes |
| Fonseca-Silva et al 2015 |  |  |  |  |  | Yes |  |  | Yes |  |
| Mai et al 2015 |  |  |  |  |  |  |  |  | Yes |  |
| Rizk et al 2014 |  | Yes |  |  |  |  | Yes |  | Yes | Yes |
| Assolini et al 2020 |  | Yes | Yes |  |  | Yes | Yes |  | Yes | Yes |
| Zeouk et al 2020 |  | Yes | Yes |  |  | Yes |  |  |  |  |
| Oliveira et al 2021 |  |  |  |  |  |  |  |  | Yes | Yes |
| Fadel et al 2019 |  |  |  |  |  |  |  |  | Yes | Yes |
| Araújo et al 2019 |  |  | Yes |  |  | Yes | Yes |  | Yes | Yes |
| Cabanillas et al 2014 |  | Yes | Yes | Yes |  |  | Yes |  |  | Yes |
| Dal Picolo et al 2014 |  |  | Yes |  |  |  |  |  | Yes | Yes |
| Ribeiro et al 2014 |  |  |  |  |  |  | Yes | Yes | Yes | Yes |
| Wong el al 2014 |  |  |  |  |  |  |  |  |  | Yes |
| Lage et al 2013 |  |  | Yes |  |  |  |  |  | Yes | Yes |
| Manjolin et al 2013 |  |  |  |  |  |  |  | Yes |  |  |
| Gervazoni et al, 2018 |  | Yes |  |  |  |  |  | Yes | Yes | Yes |
| Fabri et al 2009 |  |  |  |  |  |  |  |  | Yes |  |
| Silva et al 2011 |  |  |  |  |  | Yes |  |  | Yes |  |
| Gontijo et al 2012 |  |  |  |  |  |  | Yes |  | Yes | Yes |
| Grecco et al 2012 |  |  |  |  |  |  |  |  |  | Yes |
| Machado et al 2007 |  |  |  |  |  |  |  |  | Yes |  |
| Pereira et al 2011 |  |  |  |  |  |  |  |  | Yes | Yes |
| Salvador et al 2009 |  |  |  |  |  |  |  |  | Yes |  |
| Taled-Contini et al 2004 |  |  |  |  |  |  |  |  | Yes |  |
| Lessa et al 2024 |  |  |  |  | Yes |  |  |  | Yes |  |
| Inacio et al 2013 |  |  |  |  |  |  |  | Yes |  | Yes |
| Salvador et al 2002 |  |  |  |  |  |  |  |  |  | Yes |
| Clavin et al 2007 |  |  |  |  |  |  |  |  |  |  |
