# Supplementary material for: Flavonoids in the treatment of Leishmania amazonensis: a review of efficacy and mechanisms
Source: Front Pharmacol. 2025 Aug 7;16:1642005. doi: 10.3389/fphar.2025.1642005 (PMC12367659; doi:10.3389/fphar.2025.1642005)
Supplement: Supplementary file 5 [file Table3.docx]

Supplementary Table – S3: Characterization of flavonoids with IC_50_ and the activity level of the flavonoids for *L. amazonensis* amastigote and promastigote assays.

* ND: Not Demonstred.

|  | Characterized flavonoid | IC50 amastigote | IC50 promastigote | Activity of isolated flavonoids amastigote assay | Activity of isolated flavonoids promastigote assay |
| --- | --- | --- | --- | --- | --- |
| Dutra et al 2023 | 7,8,3′-trihydroxy-4′-methoxyisoflavone | ND | ND | ND | ND |
|  | Calycosin | ND | 4.58 ± 0.26 μg/mL (16.11 ± 0.9 μM) | ND | High |
|  | Formononetin | ND | 30.04 ± 2.09 μg/mL. (112.0 ± 7.8 μM) | ND | Moderate |
|  | Biochanin | ND | ND | ND | ND |
|  | Atalantoflavone (Erythrina sigmoidea) | 1.211±0.168μg/mL (3.6 ± 0.5 μM) | ND | High | ND |
| Araújo et al 2022 | (−)-duartin | ND | 2.47 ± 0.92 μg/mL | ND | High |
|  | (3R)-claussequinone | ND | 37.15 ± 2.43 μg/mL | ND | Moderate |
| Silva et al 2022 | Amentoflavone | ND | 7.96 ± 1.23 μg/mL | ND | High |
| Rizk et al 2022 | Amentoflavone | ND | 15.6 ± 1.1 μg/mL | ND | Moderate |
| Silva et al 2021 | luteolin | 11.78 ± 1.24 μg/mL | 31.61± 1.13 μg/mL | Moderate | Moderate |
|  | apigenin | ND | 45.6 ± 1.08 μg/mL | ND | Moderate |
| Silva et al 2021 | carajurin | 7.065 ± 1.19 µg/mL | 3.66 ± 1.16 µg/mL | High | High |
| Rizk et al 2021 | Amentoflavone | 1.24±0.50μg/mL (2.3 ± 0.93 μM) | ND | High | ND |
| Morais et al 2020 | hemileiocarpin | 1.13 ±1.4 μg/mL (compound 3) | 4.5 ± 0.5 μg/mL (compound 3) | High | High |
|  | herein | ND | ND | ND | ND |
|  | connarin | ND | ND | ND | ND |
| Silva et al 2019 | Abyssinone IV (Erythrina sigmoidea) | 4.940 ± 0.399μg/mL ( 14.7 ± 1.2 μM) | ND | High | ND |
|  | Atalantoflavone (Erythrina sigmoidea) | 1.211±0.168μg/mL (3.6 ± 0.5 μM) | ND | High | ND |
|  | Eriodictyol (Vernonanthura tweedieana) | ND | ND | ND | ND |
| Rocha et al 2019 | Brachydin A (dimeric flavonoid) | 10.45 μg/mL (20 μM) | 10.45 μg/mL (20 μM) | Moderate | Moderate |
|  | Brachydin B (dimeric flavonoid) | 1.18±0.05 μg/mL (2.20 ± 0.09 μM) | 4.91±0.54μg/mL (9.16 ± 1 μM) | High | High |
|  | Brachydin C (dimeric flavonoid) | 3.17±0.65 μg/mL (6.25 ± 1.28 μM) | 5.07±0.41μg/mL (10 ± 0.8 μM) | High | High |
| Emiliano_&_Almeida-Amaral 2018 | Apigenin | 1.04 μg/mL (3.85 μM) | ND | High | ND |
| Fonseca-Silva et al 2016 | Apigenin | 4.3 𝜇g/mL | ND | High | ND |
| Fonseca-Silva et al 2015 | Apigenin | ND | 23.7 𝜇g/mL | ND | Moderate |
| Mai et al 2015 | *G. oudiepe 1) 5,7-dihydroxy-3,3,4,6-tetramethoxyflavone* | ND | 3.05 𝜇g/mL (8.15 µM) | ND | High |
|  | 2) 30,5,7-trihydroxy-3,4,5,6-tetramethoxyflavone | ND | 3.99 𝜇g/mL (10.23 µM) | ND | High |
|  | *G. urvillei 3) 5,7-dihydroxy-3,3,4,5,6-pentamethoxyflavone* | ND | 3.9𝜇g/mL (9.65 µM) | ND | High |
|  | 4) 5,7-dihydroxy-3,3,4,5,6-pentamethoxyflavone | ND | 9.1 3.9𝜇g/mL ( 21.77 µM) | ND | High |
|  | 5) 5,7-dihydroxy-3,3,4,5,6-pentamethoxyflavone | ND | 13.66 μg/mL (31.61 µM) | ND | Moderate |
|  | 6) 40,5,7-trihydroxy-3,6,8-trimethoxyflavone | ND | 9.84 μg/mL (27.33 µM) | ND | High |
|  | 7) 40,5,7-trihydroxy-3,6-dimethoxyflavone | ND | 2.67 μg/mL (8.07 µM) | ND | High |
|  | 8) 5,7-dihydroxy-3,4,6-trimethoxyflavone | ND | 21.73 μg/mL (63.15 µM) | ND | Moderate |
|  | 9) 5,7-dihydroxy-3,4,6-trimethoxyflavone | ND | 5.3 μg/mL (14.80 µM) | ND | High |
|  | 10) 5,7-dihydroxy-3,4,6-trimethoxyflavone | ND | 13.83 μg/mL (37.17 µM) | ND | Moderate |
|  | 11) Comercial kaempferol | ND | 8.27 μg/mL (27.56 µM) | ND | High |
|  | 12) 3-methoxy-kaempferol (kaempferol-3-monomethylether) | ND | 4.49 μg/mL (14.29 µM) | ND | HIgh |
|  | 13) 3-methoxy-kaempferol (kaempferol-3-monomethylether) | ND | 7.53μg/mL (22.95 µM) | ND | High |
|  | 14) 3-methoxy-kaempferol (kaempferol-3-monomethylether) | ND | 9,45μg/mL (33.00 µM) | ND | High |
|  | 15) Semi-synthesis (triacetyl derivative) | ND | 5.57 μg/mL (12.27 µM) | ND | High |
|  | 16) Semi-synthesis (tetraacetyl derivative) | ND | 14.56 μg/mL (48.52 µM) | ND | Moderate |
| Rizk et al 2014 | *Selaginella sellowii* hydroethanolic extract (SSHE) | 20.2 μg/mL | ND | Moderate | ND |
|  | Amentoflavone | 0.1 ± 0.2 𝜇g/mL | ND | High | ND |
|  | Robustaflavone | 2.8 ± 5.3 𝜇g/mL | ND | High | ND |
| Assolini et al 2020 | 4-nitrochalcone (4NC) (comercial) | 1.02 μg/mL (4.04 μM) | 5.37μg/mL (21.2 μM) | High | High |
|  | Kaempferol 7-*O*-methyl ether | ND | 30.03 μg/mL (100 μM) | ND | Moderate |
|  | Kaempferol 3,7- di-*O*-methyl ether | 3.30±0.79μg/mL (10.5±2.5 μM) | 17.04±0.69μg/mL (54.2± 2.2 μM) | High | Moderate |
|  | Myricetin 3,7,3',4'-tetra-*O* | ND | 36.03 μg/mL (100 μM) | ND | Moderate |
|  | Gossypetin 3,7,8,4'-penta-O-methyl ether | ND | 33.23 μg/mL (100 μM) | ND | Moderate |
| Dal Picolo et al 2014 | Adunchalcone | ND | 97 ± 0.2 µg/µL (11.03 ± 2.11 µM) | ND | Moderate |
|  | Brachydin B | 1.18±0.05μg/mL (2.20 ± 0.09 µM) | 4.91±0.54μg/mL (9.16 ± 1 µM) | High | High |
|  | Brachydin C | 3.17±0.65μg/mL (6.25 ± 1.28 µM) | 5.07±0.41μg/mL (10 ± 0.80 µM) | High | High |
| Lage et al 2013 | quercetin 3-O-methyl ether | ND | 2.56±0.47μg/mL (8.1 ± 1.5 µM) | ND | High |
|  | strychnobiflavone | ND | 2.02±0.13μg/mL (3.2 ± 0.2 µM) | ND | HIgh |
| Manjolin et al 2013 | Isoquercitrin | 1,76μg/mL (3.8 µM) | ND | High | ND |
|  | Quercitrin | 4,48μg/mL (10 µM) | ND | High | ND |
|  | 7,8-dihydroxyflavone | 3,05 ± 0,25 µg/mL (12 ± 1 µM) | ND | High | ND |
|  | Orientin | 7,17 ± 0,90 µg/mL (16 ± 2 µM) | ND | High | ND |
|  | Isoorientin | 4.04±0.45μg/mL (9 ± 1 µM) | ND | High | ND |
|  | Fisetin | 0,37 ± 0,09 µg/mL (1.3 ± 0.3 µM) | ND | High | ND |
|  | Quercetin | 1,30 µg/mL (4.3 µM) | ND | High | ND |
|  | Luteolin | 2,58 ± 0,29 µg/mL (9 ± 1 µM) | ND | High | ND |
|  | Kaempferol | 14,31 µg/mL (50 µM) | ND | Moderate | ND |
|  | Galangin | 27,02 µg/mL (100 µM) | ND | Moderate | ND |
| Gervazoni; Ozório and Amaral, 2018 | 2’-Hydroxyflavanone | 0,74 µg/mL (3.09 μM) | 5,04μg/mL (20.96 μM) | High | High |
| Silva et al 2011 | Quercetin | Not determined | 9,49 µg/mL (31.4 µM) | High | High |
| Gontijo et al 2012 | 1) morelloflavone-4'''O-b-D-glycosyl | Not determined | 0.0285 μg/mL (0.0513 µM) | high | High |
|  | 2) (±)-fukugiside | Not determined | 0.0320 μg/mL (0.0446 µM) | High | High |
|  | 3) morelloflavone | 0.161μg/mL (0.29 µM) | 0.0774μg/mL (0.139 µM) | High | High |
| Grecco et al 2012 | Naringenin | ND | ND | ND | ND |
|  | sakuranetin | 51.89 μg/mL | 52.60 μg/mL | Moderate | Low |
| Salvador et al 2009 | 1) Pinostrobin | 0.0838μg/mL (0.31 μM) | ND | High | ND |
|  | 2) Pinocembrin | 0.884μg/mL (3.45 μM) | ND | High | ND |
|  | 3) Tectochrysin | 0.150μg/mL (0.56 μM) | ND | High | ND |
|  | 4) Galangin 3-methyl ether | 0.822 μg/mL (2.89 μM) | ND | High | ND |
| Lessa et al 2024 | Naringenin | Not determined | 59,87 µg/mL (219.86 μM) | ND | Low |
