# Supplementary material for: Flavonoids in the treatment of Leishmania amazonensis: a review of efficacy and mechanisms
Source: Front Pharmacol. 2025 Aug 7;16:1642005. doi: 10.3389/fphar.2025.1642005 (PMC12367659; doi:10.3389/fphar.2025.1642005)
Supplement: Supplementary file 6 [file Table4.docx]

Supplementary Table – S4: Characterization of flavonoids with CC_50_ and the SI value of the flavonoids for *L. amazonensis* amastigote and promastigote assays.

* ND: Not Demonstred.

|  |  |  |  |  |
| --- | --- | --- | --- | --- |
|  | Characterized flavonoid | CC50 | SI (seletivity index) amastigote | SI (seletivity index) promastigote |
| Dutra et al 2023 | 7,8,3′-trihydroxy-4′-methoxyisoflavone | ND | ND | ND |
|  | Calycosin | ND | ND | ND |
|  | Formononetin | ND | ND | ND |
|  | Biochanin | ND | ND | ND |
|  | Atalantoflavone (Erythrina sigmoidea) | 14.82±1.65μg/mL (44.1 ± 4.9 μM) | 12.12 | ND |
| Araújo et al 2022 | (−)-duartin | 346.41 ± 40.99 μg/mL | ND | 9.3 |
|  | (3R)-claussequinone | 387.79 ± 25.93 μg/mL | ND | 157 |
| Silva et al 2022 | Carajurin | 258.2 ± 1.20 μg/mL | ND | 32.4 |
| Rizk et al 2022 | amentoflavone | ND | ND | ND |
| Silva et al 2021 | luteolin | 8.005 ± 1.23 μg/mL | 0.679 | ND |
|  | apigenin | 11.87 ± 1.32 μg/mL | ND | ND |
| Silva et al 2021 | carajurin | 16.48 ± 1.10 (µg/mL) | 34.8 | ND |
| Rizk et al 2021 | Amentoflavone | 12.00 μg/mL (22.3 µM) | greater than 10 | ND |
| Morais et al 2020 | hemileiocarpin | 7.2 μg/mL (compound 3) | 6.3 | ND |
|  | herein | ND | ND | ND |
|  | connarin | ND | ND | ND |
| Silva et al 2019 | Abyssinone IV (Erythrina sigmoidea) | 31.32±0.94μg/mL ( 79.8 ± 2.4μM) | 5.43 | ND |
|  | Atalantoflavone (Erythrina sigmoidea) | 14.82±1.65μg/mL (44.1 ± 4.9 μM) | 12.12 | ND |
|  | Eriodictyol (Vernonanthura tweedieana) | ND | ND | ND |
| Rocha et al 2019 | Brachydin A (dimeric flavonoid) | 10.45 μg/mL (20 μM) | ND | ND |
|  | Brachydin B (dimeric flavonoid) | 10.73 μg/mL (20 μM) | 9.1 | ND |
|  | Brachydin C (dimeric flavonoid) | 10.13 μg/mL (20 μM) | 3.2 | ND |
| Emiliano_&_Almeida-Amaral 2018 | Apigenin | ND | ND | ND |
| Fonseca-Silva et al 2016 | Apigenin | ND | ND | ND |
| Fonseca-Silva et al 2015 | Apigenin | ND | ND | ND |
| Mai et al 2015 | *G. oudiepe 1) 5,7-dihydroxy-3,3,4,6-tetramethoxyflavone* | ND | ND | ND |
|  | 2) 30,5,7-trihydroxy-3,4,5,6-tetramethoxyflavone | ND | ND | ND |
|  | *G. urvillei 3) 5,7-dihydroxy-3,3,4,5,6-pentamethoxyflavone* | ND | ND | ND |
|  | 4) 5,7-dihydroxy-3,3,4,5,6-pentamethoxyflavone | ND | ND | ND |
|  | 5) 5,7-dihydroxy-3,3,4,5,6-pentamethoxyflavone | ND | ND | ND |
|  | 6) 40,5,7-trihydroxy-3,6,8-trimethoxyflavone | ND | ND | ND |
|  | 7) 40,5,7-trihydroxy-3,6-dimethoxyflavone | ND | ND | ND |
|  | 8) 5,7-dihydroxy-3,4,6-trimethoxyflavone | ND | ND | ND |
|  | 9) 5,7-dihydroxy-3,4,6-trimethoxyflavone | ND | ND | ND |
|  | 10) 5,7-dihydroxy-3,4,6-trimethoxyflavone | ND | ND | ND |
|  | 11) Comercial kaempferol | ND | ND | ND |
|  | 12) 3-methoxy-kaempferol (kaempferol-3-monomethylether) | ND | ND | ND |
|  | 13) 3-methoxy-kaempferol (kaempferol-3-monomethylether) | ND | ND | ND |
|  | 14) 3-methoxy-kaempferol (kaempferol-3-monomethylether) | ND | ND | ND |
|  | 15) Semi-synthesis (triacetyl derivative) | ND | ND | ND |
|  | 16) Semi-synthesis (tetraacetyl derivative) | ND | ND | ND |
| Rizk et al 2014 | *Selaginella sellowii* hydroethanolic extract (SSHE) | ND | Fibroblast cells (NIH/3T3) (12.2); Murine macrophages (J774.A1) (8.2) | ND |
|  | Amentoflavone | ND | NIH/3T3 (22); J774.A1 (30) | ND |
|  | Robustaflavone | ND | NIH/3T3 (9.1); J774.A1 (1.1) | ND |
| Assolini et al 2020 | 4-nitrochalcone (4NC) (comercial) | 2.21μg/mL (8.73 μM) | 2.1 | 0.41 |
|  | Kaempferol 7-*O*-methyl ether | 30.03 μg/mL (100μM) | ND | ND |
|  | Kaempferol 3,7- di-*O*-methyl ether | 31.43 μg/mL (100 μM) | ND | 1.84 |
|  | Myricetin 3,7,3',4'-tetra-*O* | 36.03 μg/mL (100 μM) | ND | ND |
|  | Gossypetin 3,7,8,4'-penta-O-methyl ether | 33.23 μg/mL (100 μM) | ND | ND |
| Dal Picolo et al 2014 | Adunchalcone | 27.21±3.65μg/mL (53.71 ± 7.21 µM) | ND | 4.86 |
|  | Brachydin B | 10.73 μg/mL (20 µM) | 9.1 | ND |
|  | Brachydin C | 10.13μg/mL (20 µM) | 3.2 | ND |
| Lage et al 2013 | quercetin 3-O-methyl ether | 62.93±8.19μg/mL (199.0 ± 25.9 µM) | ND | 10.4 |
|  | strychnobiflavone | 78.81±2.84μg/mL (125 ± 4.5 µM) | ND | 24.6 |
| Manjolin et al 2013 | Isoquercitrin | ND | ND | ND |
|  | Quercitrin | ND | ND | ND |
|  | 7,8-dihydroxyflavone | ND | ND | ND |
|  | Orientin | ND | ND | ND |
|  | Isoorientin | ND | ND | ND |
|  | Fisetin | ND | ND | ND |
|  | Quercetin | ND | ND | ND |
|  | Luteolin | ND | ND | ND |
|  | Kaempferol | ND | ND | ND |
|  | Galangin | ND | ND | ND |
| Gervazoni; Ozório and Amaral, 2018 | 2’-Hydroxyflavanone | 21,18 µg/mL (88.15 ± μM)  ) | 28.5 (Wilt type) 26.2 (antimony resistent) | ND |
| Silva et al 2011 | Quercetin | ND | ND | ND |
| Gontijo et al 2012 | 1) morelloflavone-4'''O-b-D-glycosyl | ND | ND | ND |
|  | 2) (±)-fukugiside | ND | ND | ND |
|  | 3) morelloflavone | 0.161μg/mL (0.29 µM) | ND | ND |
|  | 4) Morelloflavone-7,4,7,3,4-penta-O-acetyl | 0.211μg/mL (0.3800 µM) | ND | ND |
|  | 5) Morelloflavone-7,4',7''',3''',4'''-penta-O-methyl | 0.211μg/mL (0.3800 µM) | ND | ND |
|  | 6) Morelloflavone-7,4',7''',3''',4'''-penta-O-butanoyl | 0.211μg/mL (0.3800 µM) | ND | ND |
| Grecco et al 2012 | Naringenin | ND | ND | ND |
|  | sakuranetin | 39.50 μg/mL | ND | ND |
| Salvador et al 2009 | 1) Pinostrobin | ND | ND | ND |
|  | 2) Pinocembrin | ND | ND | ND |
|  | 3) Tectochrysin | ND | ND | ND |
|  | 4) Galangin 3-methyl ether | ND | ND | ND |
| Lessa et al 2024 | Naringenin | ND | ND | ND |
