# Supplementary material for: Flavonoids in the treatment of Leishmania amazonensis: a review of efficacy and mechanisms
Source: Front Pharmacol. 2025 Aug 7;16:1642005. doi: 10.3389/fphar.2025.1642005 (PMC12367659; doi:10.3389/fphar.2025.1642005)
Supplement: Supplementary file 7 [file Supplementaryfile2.docx]

Supplementary Chart – S2 Chemical structure of the most active flavonoid against promastigote forms

|  | Characterized flavonoid | Chemical Structure | Pubchem CID |
| --- | --- | --- | --- |
| Dutra et al 2023 | Calycosin | 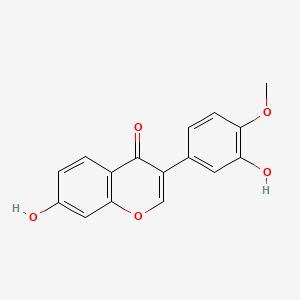 | CID 5280448 |
| Araújo et al 2022 | Duartin (-) | 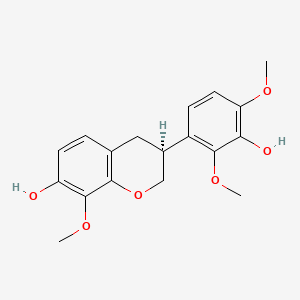 | CID  6710727 |
| Silva et al 2021 | Carajurin | 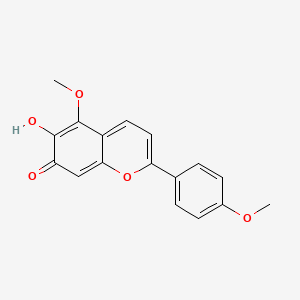 | CID 9948177 |
| Morais et al 2020 | **Hemileiocarpin** | 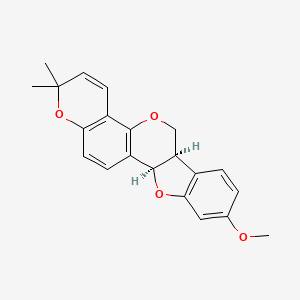 | **CID 70995758** |
| Rocha et al 2019  Dal Picolo et al 2014 | Brachydin B (dimeric flavonoid) | 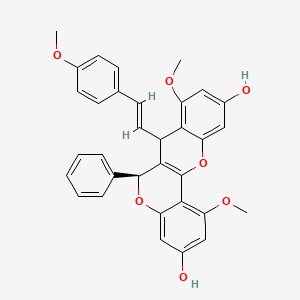 | SID 275575602 |
|  | Brachydin C (dimeric flavonoid) | 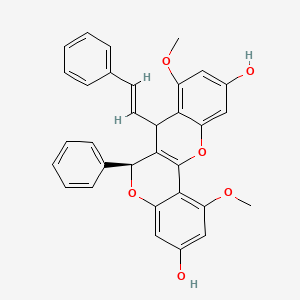 | SID 275575601 |
| Assolini et al 2020 | 4-nitrochalcone | 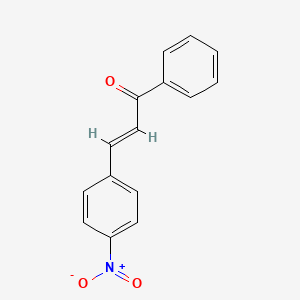 | CID 5377323 |
| Lage et al 2013 | Strychnobiflavone | 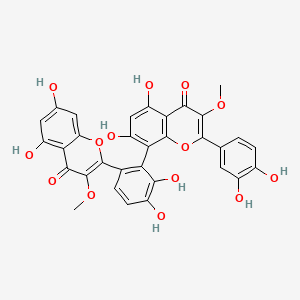 | CID 5491475 |
| Gervazoni; Ozório and Amaral, 2018 |  |  |  |
|  | 2’ Hydroxyflavanone | 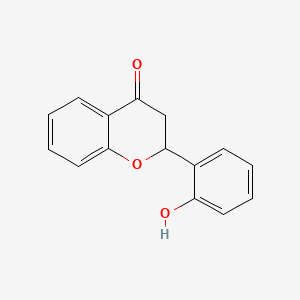 | CID 176925 |
| Silva et al 2011 | Quercetin | 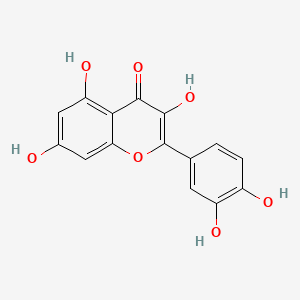 | CID 5280343 |
| Gontijo et al 2012 | (+/-)-Fukugiside | 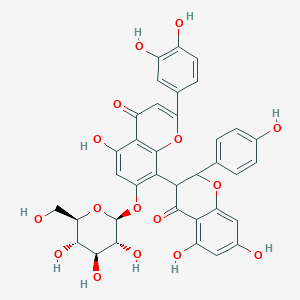 | CID 11968471 |
|  | Morelloflavone | 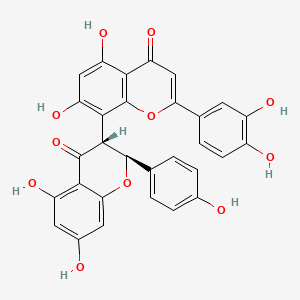 | CID 5464454 |
